# Low migratory connectivity and similar migratory strategies in a shorebird with contrasting wintering population trends in Europe and West Africa

**DOI:** 10.1038/s41598-024-55501-y

**Published:** 2024-02-28

**Authors:** Teresa Catry, Edna Correia, Jorge S. Gutiérrez, Pierrick Bocher, Frédéric Robin, Pierre Rousseau, José P. Granadeiro

**Affiliations:** 1 Centro de Estudos do Ambiente e do Mar (CESAM), Departamento de Biologia Animal, Faculdade de Ciências da Universidade de Lisboa, 1749-016 Lisbon, Portugal; 2 Departamento de Anatomía, Biología Celular y Zoología, Facultad de Ciencias, Universidad de Extremadura, Badajoz, Spain; 3 Ecología en el Antropoceno, Unidad asociada CSIC-UEX, Universidad de Extremadura, Badajoz, Spain; 4 Laboratory Littoral Environnement et Sociétés UMR LIENSs 7266 CNRS-La Rochelle University, La Rochelle, France; 5 Ligue pour la Protection des Oiseaux (LPO), Rochefort, France; 6 National Nature Reserve of Möeze-Oléron, Ligue pour la Protection des Oiseaux (LPO), Saint-Froult, France

**Keywords:** Ecology, Animal migration, Conservation biology

## Abstract

Migratory shorebird populations are declining worldwide, showing an apparent inability to respond to the interplaying challenges emerging along their flyways. Within the East Atlantic Flyway, non-breeding populations show moderate to strong declines in Sub-Saharan Africa, contrasting with stable or increasing trends in Europe. Local factors are insufficient to explain the opposite tendencies and, therefore, investigating migratory strategies and connectivity of these populations may help identifying the drivers of their demography. We followed the migratory journeys of 20 grey plovers (*Pluvialis squatarola*) from their wintering grounds in Guinea-Bissau (West Africa), Portugal and France (Europe) using tracking devices. Grey plovers wintering in Africa and Europe were found to share breeding grounds at European Russia and Western Siberia, revealing low migratory connectivity in the Eastern Atlantic population. All individuals followed a “skipping” migratory strategy, flying mostly mid-distance bouts, and using an unexpected large network of stopover sites to re-fuel usually for short periods. We identified 66 different stopover sites along the West African, European and Russian/Siberian coasts. All birds stopped at the Wadden Sea in both migratory periods, highlighting the importance of this region and the risk for a potential bottleneck. Low migratory connectivity and similar migratory strategies shared by grey plovers wintering in Europe and West Africa do not support their contrasting population trends.

## Introduction

Shorebirds, or waders, are renowned for their impressive long-distance migrations between high-latitude breeding areas and temperate and tropical coastal wintering grounds. To accomplish these extraordinary flights, they rely on a network of sites that act as refuelling stops—the stopover sites^1,2^. Many shorebirds are facing population declines at flyway or global scales, mainly as a result of global changes induced by human activities^3–7^. Global warming, which can result in a phenological mismatch triggered by the inability of shorebirds to keep pace with advancing snowmelt at their breeding areas, along with habitat loss and the potential disruption of the network of suitable wetlands that shorebirds rely on throughout their annual cycle, are of particular concern^4,8,9^.

The population trends of several migratory shorebird species in the East Atlantic Flyway (EAF) suggest that, despite facing global threats, distinct populations may be impacted differently. Indeed, a current pattern of mild to moderate increases in non-breeding populations from Europe strongly contrasts with moderate to strong declines in Africa, southward from Mauritania and Senegal^6^. It may seem counterintuitive that there are increases in European populations, given the well-documented human-driven habitat loss and deterioration in several important wetlands in this continent (e.g.^10–12^). In contrast, key areas of the flyway in West Africa, such as the Banc d’Arguin (Mauritania) and the Bijagós Archipelago (Guinea-Bissau) show less evident signs of habitat degradation^6^. While long-term research at some of these sites is beginning to partly reveal the causes of local population declines of some species (see^13^), flyway-scale studies are critical to establish the links between the often geographically separated causes and consequences that explain the changing numbers of shorebirds (e.g.^14,15^). Effects of global warming at Arctic breeding grounds, for instance, are now starting to be detected at non-breeding areas. Malnutrition in early life due to Arctic warming has led to body shrinkage in red knots (*Calidris canutus*), reducing individual fitness and survival in their main tropical wintering area in West Africa^14^. Likewise, the need to keep pace with premature springs in Siberia has forced bar-tailed godwits (*Limosa lapponica*) to shorten their refuelling period in the Wadden Sea, resulting in lower individual survival, especially in years of low prey availability^15^. Changes at intermediate stopover sites can indeed have profound impact in shorebird numbers at their final destinations^15^. For instance, the deterioration of feeding conditions at the Dutch Wadden Sea, a stopover area for red knots migrating between Siberia and West Africa, has been identified as one cause of population declines at Banc d’Arguin^11^.

Following the pattern described for several migratory shorebirds in the EAF, non-breeding populations of Grey Plover (*Pluvialis squatarola*) show a stable trend in Europe and moderate to strong declines in Africa^6^. Around two hundred thousand grey plovers (from the nominate subspecies, *Pluvialis squatarola squatarola,* hereafter designated as Eastern Atlantic population) breed in Siberia and spend the non-breeding season in the west coasts of Europe and Africa. Major wintering concentrations are found in The Netherlands, France and UK (Europe), and Mauritania, Guinea-Bissau and Guinea (Africa;^2^). Grey plovers have shown an apparent dramatic decline in one of the most pristine areas within the Flyway, the Bijagós Archipelago, in Guinea-Bissau, with numbers falling from ca. 40,000 birds during the late eighties and early nineties^16,17^, to ca. 10,000 birds since 2017^6^. Despite the growing research efforts during the last decade focused on shorebirds and their habitats in Guinea-Bissau (e.g.^18–22^), there is no evidence of a strong local effect explaining these declines.

Grey plovers from the Eastern Atlantic population are thought to breed in Arctic Russia, as far east as the Taimyr Peninsula, located at 80–90°E^2^. However, it remains unclear whether this population follows a leap-frog or a chain migration pattern, i.e., if northern breeding populations winter further south than more southerly breeders, or if the latitudinal distribution of wintering grounds follows that of breeding areas^1^. Biometric studies suggested that grey plover populations might be segregated in the Arctic breeding grounds, with short-billed birds occurring in Western Siberia (west to Yamal Peninsula) and long-billed birds in Central (west to Taimyr) and Eastern (east of Taimyr) Siberia^23^. Based on bill size, Wymenga et al.^24^ suggested that grey plovers wintering in Guinea-Bissau breed mostly from Central and possibly Eastern Siberia, but no tracking or ringing data have confirmed this hypothesis. Recent but still preliminary tracking data suggests that the low-Arctic breeding grounds of Kolguev Island (49°E) are linked to West Africa, and that several non-breeding areas in Europe are connected to Western and Central Siberia^25^.

In this study, we describe the migratory journeys of grey plovers tagged with Argos PTT and GPS trackers at three different wintering sites: Guinea-Bissau in West Africa, and Portugal and France in Europe. First, we aimed at determining migratory connectivity for the Grey Plover Eastern Atlantic population, which involves geographically and temporally linking populations among different stages of the annual cycle. Such information is decisive to identify potential bottlenecks along the flyway and, importantly, understand whether populations wintering in Europe and West Africa are under the same environmental influences. We expect that under a low migratory connectivity scenario, i.e., if grey plovers wintering over a large area across Europe and Africa mix at a particular breeding area, unfavourable breeding conditions are unlikely to explain the contrasting population trends observed in Europe and Africa. Likewise, if different wintering populations use the same stopover areas, habitat deterioration or loss at shared areas would tend to affect them equally. In a high migratory connectivity scenario, in which grey plover populations are segregated during both the breeding and non-breeding seasons, contrasting trends in Europe and Africa may have been driven by conditions experienced during breeding. The second goal of this study was to compare the migratory phenology and strategies of Grey Plover wintering in Europe and Africa, also trying to find if differences in these traits could provide some cues on the divergent population trends. Several Arctic breeding shorebirds wintering in West Africa have been suggested to follow a “jumping” strategy during northward migration, flying long non-stop flights with only one (sometimes two) stop(s) for refuelling, which could potentially allow a faster journey while also avoiding less suitable refuelling areas, parasites and predators (e.g.^1,15,26,27^). It is currently unknown whether grey plovers that winter in West Africa follow such a strategy, which would contrast with the idea of a journey with medium-to-short flights and more frequent stops (“skipping” and “hopping” strategies, respectively^28^) in plovers departing from European grounds.

## Methods

For the sake of simplicity, and given that the study is carried on the northern hemisphere, northward and southward migration will be referred to as spring and autumn migration, respectively, while summer and winter will be used to refer breeding and non-breeding (outside migration) seasons.

### Bird capture and transmitter deployment

Grey plovers were captured at three major non-breeding sites of the EAF, (1) the Bijagós Archipelago, in Guinea-Bissau, (2) the Tagus estuary, in Portugal, and (3) the Pertuis Charentais, in France. Birds were mist-netted, during the night, at roosting sites. Before fitting the transmitter, all birds were ringed and their body mass (to the nearest g), wing length (maximum chord, to the nearest mm), and age (1st year–juvenile, 2nd year, adult^29,30^) were recorded. Three different models of transmitters were used in this study. In Guinea-Bissau (hereafter GB), 14 birds were captured during the non-breeding seasons of 2018/2019 and 2019/2020, on three different islands/islets of the Bijagós Archipelago (Imbone, Bubaque and Formosa). Eight of these birds were fitted with 5g Argos PTT (Microwave Telemetry Inc., USA) and the remaining five with 6g PinPoint GPS Argos transmitters (Lotek Wireless Inc., Canada), both models incorporating solar panels. Four grey plovers were captured and tagged with PinPoint GPS Argos transmitters in Portugal, two in spring 2019 and two in the autumn/winter 2020. One of the birds tagged in spring in Portugal was later confirmed to winter in Guinea-Bissau. In France, three individuals were fitted with 6g GPS-UHF transmitters (PICA UHF-SRD with solar charger, Ecotone, Gdansk, Poland) during the wintering seasons of 2018/2019 and 2019/2020. Tracking devices were deployed using a leg-loop harness with a 2 mm Teflon ribbon. The weight of the transmitter and the harness always represented less than 3% of the bird body mass.

Argos PTT tags were programmed to a duty cycle of 10 h ON and 25 h OFF, with the XT option enabled, allowing the PTT to transmit during the scheduled OFF time if the battery is fully charged. PinPoint GPS Argos tags collect GPS data that are transmitted via Argos with the Pass Prediction function (once the tag has three GPS fixes it will predict when an Argos satellite will be in position to receive data and will initialize transmission during the predicted transmit window). At the time of transmission, the Argos system will use the Doppler shift method to obtain a (much less accurate) position. PinPoint GPS Argos tags were programmed to record between three and five GPS locations per day. GPS-UHF loggers were programmed to record one GPS-position every hour. For these transmitters, data from migration periods were stored in the device and downloaded when the birds returned to the study area, by using a set of reception antennas located at the main high-tide roosts.

### Processing of tracking data

The first step of processing track files involved only birds equipped with Pinpoint GPS Argos tags and consisted of removing all fixes with location class “Z” (invalid location). Then, we combined files delivered by the (lower accuracy) Argos system with those containing higher accuracy GPS fixes, using the corresponding time stamp. We detected a few fixes with the same timestamps in our dataset and, in these cases, we selected the one obtained by the GPS system, or if provided by the same system, the fix that minimized the cumulative distance between the two neighbouring points. Also, Pinpoint GPS Argos tags occasionally provided bursts of locations with very short intervals (sometimes delivering unrealistic speeds), and therefore we iteratively removed fixes until they were separated by at least 10 min. Subsequent procedures were then applied to all tracks, irrespective of the type of tag used. All maps and analysis were carried out in R 4.2.3^31^, using packages *sf*^32^, *raster*^33^, *amt*^34^ and their dependencies.

We calculated the great circle distances and the corresponding time intervals between successive locations in each track. Any fixes involving speeds higher than 30 m s^−1^ (118.8 km h^−1^) were removed from subsequent analysis. To remove unresolved, often smaller, location errors that persisted in virtually all tracks, we applied a median filter with a moving window width (k) of 3, keeping the first and last points of the track unchanged (e.g.^35^). The median filter is a smoothing procedure, approximating the value of an observation based on the median of neighbouring values^35^. We then individually analysed each track using a custom-made Shinny app (running R code) to classify all points between the start and end of migratory displacements (i.e., movements larger than location errors involving a succession of fixes, in the same direction, corresponding to a net displacement larger than 50 km). These points were classified as “travelling” and all remaining points were classified as “wintering”, “breeding” and “stopover” (stationary periods > 24 h between wintering and breeding areas), depending on their location. Some stationary periods lasted less than 24 h, and these were not considered as true “stopovers”, and were therefore classified as “stationary”.

Due to the large intervals between fixes, stopover duration may be severely underestimated. In fact, birds may arrive to the stopover well before the first fix in the area, and will often depart earlier than the first fix of the following travelling bout. To account for this situation, we first calculated the distance between the last “travelling” fix and the first fix at the stopover site. We then used the average speed recorded in the travelling bout, to calculate an “estimated time of arrival” (timestamp of the last travelling point plus time required to travel the distance to the stopover, given the previous travel speed). We used the same procedure to calculate the “estimated time of departure”, using the average travel speed recorded in the travelling bout before the next stationary point (timestamp of the first travelling fix minus time required to travel the distance to previous stopover, given the average speed of the following travel bout). To avoid large errors in these estimations, this procedure was only implemented when the fixes representing transitions between stopover and travel states were less than 24 h apart. The duration (and corresponding distance, as appropriate) of all events within the migratory route, were calculated based on these timestamped fixes. However, whenever the transition between two migratory stages involved gaps longer than 24h, the above metrics were excluded from the analysis. For representation purposes, we used a Lambert equal area projection, centred close to the Wadden Sea (at 54°N, 8°W).

Our tracking database includes complete (from breeding to wintering areas or vice-versa) and incomplete (ending before the arrival to the breeding or wintering areas) migratory journeys. Complete trips were used to estimate all migratory parameters (number and duration of stopovers, number of travelling days, migration speed, duration and distance) while incomplete trips were used to identify stopover sites and calculate other relevant parameters until the device stopped transmitting (e.g. number of stopovers before or after the Wadden Sea).

Migration duration and migration speed were calculated as the number of days elapsed between departure and arrival from/to the breeding or wintering grounds and the total distance flown (km) divided by the total time (days) spent on migration, respectively. The fact that these parameters do not account for the time spent on fuelling before the first flight^36,37^ is acknowledged.

We ran generalized linear models (GLMs) with Gaussian family (Poisson family was used for the model regarding the number of stopovers) to investigate the relationship between a set of dependent variables, namely arrival date, migration speed, number of stopovers and total duration in stopovers, with migration departure date (explanatory variable). The wintering origin of the birds (GB vs. Europe) was included as a factor in interaction with departure date to account for different effects of departure date on migratory performance according to total migratory distance and potential different migratory strategies. We performed separate models for spring and autumn migrations, as the sample size, especially for autumn migration, was low. Model selection was always performed by starting with the full model (including the interaction between departure date and wintering origin) and comparing increasingly simple nested models, successfully removing non-significant predictors and interactions, with likelihood ratio tests (F-tests). Results in the text are presented for the minimum adequate model (for full information on model results see Supplementary Table S1).

To evaluate the rate of stopover site finding according to the number of grey plovers tracked, we built accumulation curves from 100 permutations by adding birds in random order, using the function *specaccum* of the *vegan* R package^38^.

Throughout the results, metrics are reported as mean ± standard deviation, unless stated otherwise.

### Ethics declarations

Bird capture and tagging were performed in accordance with the guidelines and regulations of each of the relevant institutions: Institute for Nature Conservation and Forests, Portugal (permits 242/2019, 233/2020, 389/2019/CAPT, 793/2020/CAPT), National Museum Programme, France (permit CRBPO PP366), and Institute for Biodiversity and Protected Areas (IBAP), Guinea-Bissau. Experimental protocols (bird capture and tagging) were further approved by the Animal Welfare Body of Faculty of Sciences, University of Lisbon (ORBEA-FCUL), following the requirements of the Directive 2010/63/EU of the European Parliament and of the council for the protection of animals used for scientific purposes.

## Results

Grey plovers tagged in GB included one juvenile that over-summered in the Bijagós Archipelago, four second calendar-year (non-breeders) and eight adult (> 2nd year) birds. All individuals tagged in PT and FR were adults. One bird captured in PT during spring migration spent the wintering season in inland GB, near Cantanhez National Park. Six plovers, one from FR and five from GB, were tracked for more than one year, including three 2nd calendar year birds, two of which bred in the following year. Still, their migrations on the second year of tracking were often incomplete, as the tracking devices stopped transmitting data while the bird was *en route*. Transmitter signal loss occurred at all stages of the birds’ life cycle, with 50% recorded during wintering and breeding periods. During migration, signal loss was mostly associated to the Wadden Sea, with only three birds “lost” elsewhere (one in Russia, one in France and one in Mauritania). Still, we could not directly link transmission loss to mortality, as this loss was always abrupt, with no consecutive fixes at the same location. The number of complete and incomplete migratory events for birds from each study area is presented in Table 1.

**Table 1:** Number of grey plovers tagged and number of complete and incomplete spring and autumn migratory events of tracked birds according to the three study wintering areas (Guinea-Bissau, Portugal and France).

Tagging site	Age (at tagging)	Number of birds tagged	Tag model (n)	Complete		Incomplete	
				Spring	Autumn	Spring	Autumn
Guinea-Bissau	Adults	9 + 3^a^	Argos PTT (6) Pinpoint GPS Argos (6)	10 (8)	4 (3)	2 (2)	3 (3)
	2nd calendar year (non-breeders)	4	Argos PTT (1), Pinpoint GPS Argos (3)	4 (4)	4 (4)	0	0
	Juveniles	1	Argos PTT	0	0	0	0
Portugal	Adults	4^b^	Pinpoint GPS Argos	3 (3)	2 (2)	0	1 (1)
France	Adults	3	GPS-UHF	4 (3)	4 (3)	0	0

The number of individuals tracked in each season is presented in parentheses.

^a^Three grey plovers tracked as adults were tagged in the previous year as 2nd calendar year (non-breeders).

^b^One grey plover tagged in spring in Portugal was later confirmed to winter in Guinea-Bissau.

### Movements during the non-breeding season

During the non-breeding season grey plovers typically exhibited site fidelity, performing short movements between foraging and roosting sites within their wintering area, i.e., the Bijagós Archipelago, the Tagus estuary or Pertuis Charentais. Exceptions were recorded for one bird that left the Bijagós Archipelago (GB) in mid-March and moved 500 km south to spend the rest of the winter in Sierra Leone, and another bird tagged in France that travelled ca. 1000 km between La Rochelle and the north coast of France (near Calais) in March, returning to its wintering area five days later.

### Breeding and over-summering areas

**Figure 1:**
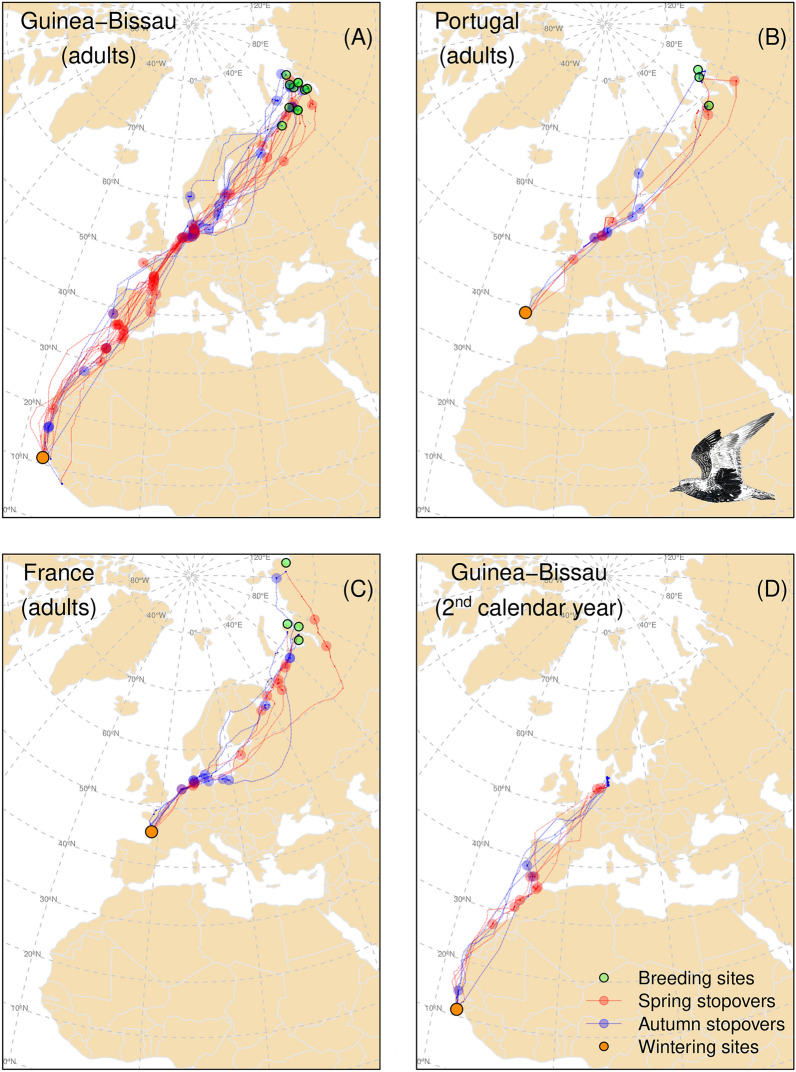
Migratory journeys of grey plovers. Migratory routes, stopover sites and breeding sites/over-summering areas of grey plovers wintering in Guinea-Bissau (**A**—adults, **D**—2nd calendar year), Portugal (**B**) and France (**C**). The map uses a Lambert equal area projection, centred close to the Wadden Sea (at 54°N, 8°W). The basemap was obtained from R package rnaturalearth (version 0.1.0. https://CRAN.R-project.org/package=rnaturalearth), running in R software, which links to Natural Earth (https://www.naturalearthdata.com/) free vector maps.

Most grey plovers (n = 10) bred on the Yamal Peninsula in Western Siberia, regardless of their tagging origin (Fig. 1). A smaller number of birds (n = 5, four from GB and one from PT) bred west of the Yamal, in European Russia, south of Pechora Sea, reaching longitudes of 50°E. Only one grey plover, tagged in France, travelled to breed on the Taimyr Peninsula in Central Siberia (Fig. 1). On average, birds spent 52.9 ± 14.0 days (range 32–86; n = 14) at the breeding grounds. Birds tracked during two consecutive breeding seasons (n = 3, one wintering in FR and two in GB) bred exactly in the same location (at the Yamal Peninsula).

Second calendar-year birds that were tagged in GB spent the summer in the Danish (n = 2) and German (n = 2) Wadden Sea, and remained within a restricted area, with a radius of less than 20 km, for an average of 99.5 ± 32.9 days (range 72–147 days).

### Migratory journeys

#### Spring migration (adults)

Grey plovers from the three study areas started spring migration mostly during the first half of May (Table 2). The earliest bird to leave GB departed on April 19th, while the latest one left on May 24th.

**Table 2:** Parameters of spring and autumn migratory journeys of adult grey plovers wintering in Guinea-Bissau, Portugal and France, as well as 2nd calendar year individuals (non-breeders) wintering in Guinea-Bissau.

	Guinea-Bissau		Portugal		France		Guinea-Bissau (2nd calendar year)	
	Spring	Autumn	Spring	Autumn	Spring	Autumn	Spring	Autumn
Departure date	**129 ± 3.2** (9 May) [127–133]	**215 ± 15** (3 Aug) [202–236]	**129 ± 3.2** (9 May) [127–133]	**222 ± 1.2** (10 Aug) [220–223]	**125 ± 21.3** (5 May) [94–140]	**207 ± 3.8** (26 Jul) [204–212]	**134 ± 5.7** (14 May) [127–140]	**266 ± 30** (23 Sep) [244–300]
Arrival date	**162 ± 9.3** (11 June) [149–176]	**298 ± 33** (25 Oct) [251–325]	**158 ± 5.5** (7 June) [152–163]	**261 ± 4.9** (18 Sep) [257–264]	**157 ± 2.1** (6 June) [155–159]	**239 ± 10.7** (27 Aug) [229–254]	**161 ± 5.9** (10 June) [155–169]	**276 ± 42** (3 Oct) [248–324]
Mean number of stopovers	**4.7 ± 1.2** [3–6]	**4.8 ± 1.3** [3–6]	**2.7 ± 1.5** [1–4]	**3.5 ± 0.7** [3–4]	**3.8 ± 1.0** [3–5]	**3.3 ± 1.5** [2–5]	**2.3 ± 0.5** [2–3]	**0.7 ± 1.2** [0–2]
Mean stopover duration (days) (n = stopovers)	**5.3 ± 3.4** [1.1–14.7] (n = 47)	**15.8 ± 24.5** [1.1–95.8] (n = 19)	**7.4 ± 7.4** [1.3–19.4] (n = 8)	**9.7 ± 11.1** [1.6–24.1] (n = 6)	**6.8 ± 12.4** [1.1–48.5] (n = 15)	**8.0 ± 6.9** [1.5 – 19.5] (n = 13)	**8.3 ± 4.9** [1.2–15] (n = 9)	**8.0 ± 3.4** [5.6–11.9] (n = 3)
Mean number of stationary periods	**2.0 ± 2.5** [0–8]	**2.0 ± 2.4** [0–5]	**0.3 ± 0.6** [0–1]	**0**	**8.3 ± 3.7** [4–13]	**3.5 ± 2.6** [1–7]	**1.0 ± 0.8** [0–2]	**1**
Mean stationary duration (days)	**0.6 ± 0.2** [0.2–1] (n = 20)	**0.6 ± 0.2** [0.3–1] (n = 8)	**0.6** (n = 1)	–	**0.3 ± 0.2** [0.0–0.8] (n = 33)	**0.4 ± 0.3** [0.1–1] (n = 14)	**0.7 ± 0.2** [0.6–1] (n = 4)	**0.3** (n = 1)
Total migration duration (days)	**33.7 ± 5.9** [24.5–39]	**83.1 ± 29.0** [45.5–116]	**28.1 ± 2.8** [24.9–30.2]	**34.4**	**32.4 ± 20.2** [18.8–62.2]	**31.9 ± 7.4** [25–42.3]	**23.9 ± 3.2** [20.3–26.8]	**9.7 ± 10.0** [3.4–21.2]
Migration distance (km)	**11,476 ± 431** [10907–12067]	**11,013 ± 392** [10578- 11436]	**8094 ± 922** [7419–9144]	**7324 ± 614** [6889–7758]	**6411 ± 306** [6198–6858]	**6632 ± 589** [6099–7466]	**7009 ± 124** [6894–7182]	**7003 ± 206** [6698–7129]
Migration speed (km/day)	**355 ± 68** [282–445]	**148 ± 59** [97–233]	**294 ± 27** [264–319]	**200**	**242 ± 99** [110–338]	**213 ± 36** [177–263]	**294 ± 38** [257–330]	**1148 ± 854** [328–2139]

Mean ± SD are presented in bold and range in square brackets. Sample size (n) for stopover (i.e., stops > 24h) and stationary (i.e., stops < 24h) duration is also given in parenthesis. Departure and arrival dates refer to the first and last days of migration and are expressed as the number of days elapsed since the 1st of January. Only complete migrations were considered (see sample size in Table 1).

**Figure 2:**
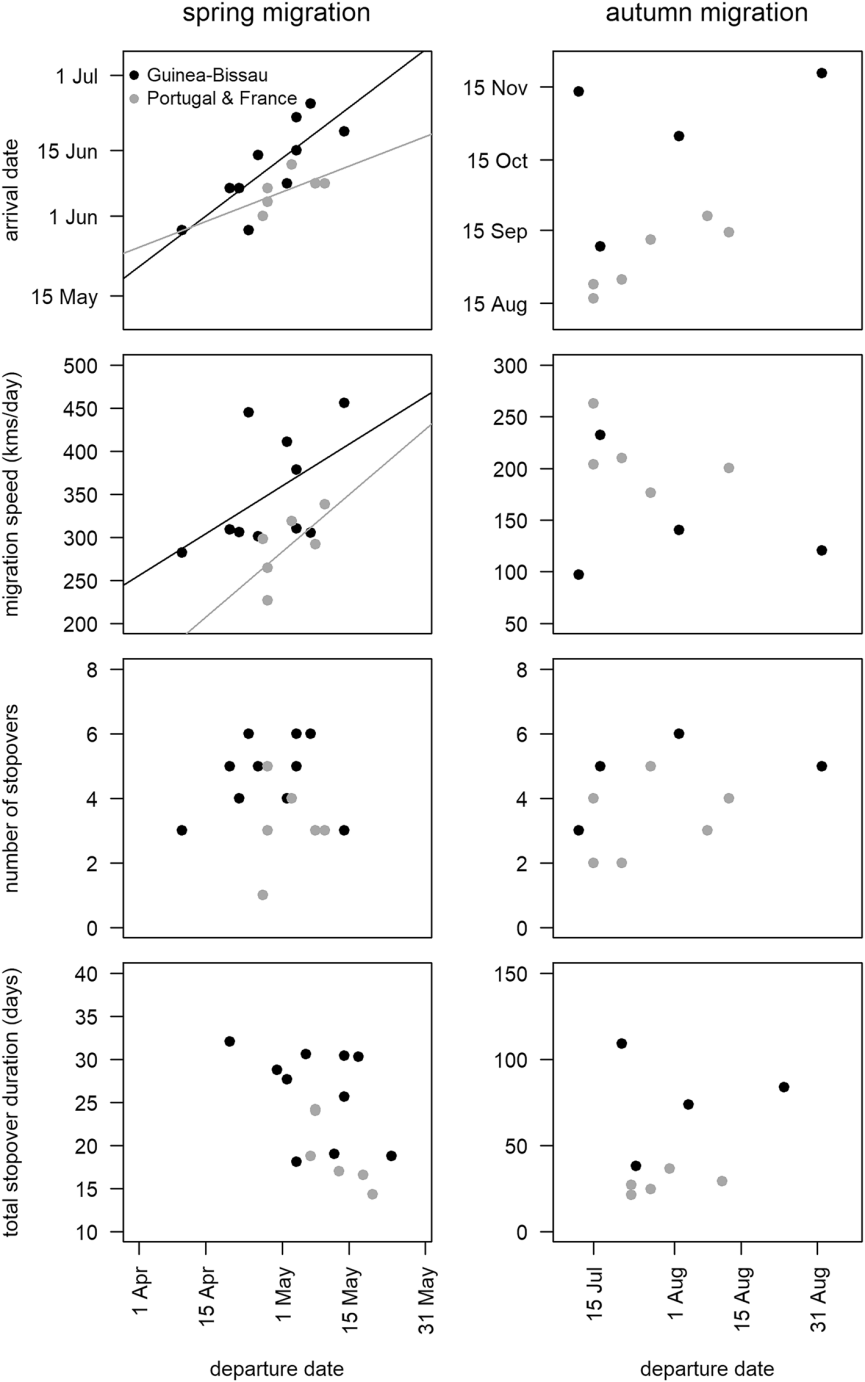
Relationship between departure date and (i) arrival date, (ii) migration speed (kms/day), (iii) number of stopovers and (iv) total duration in stopover of grey plovers wintering in Guinea-Bissau and Europe (Portugal and France) during spring and autumn migration. Significant (p < 0.05) relationships derived from generalized linear models (with the wintering origin included as a factor; see Supplementary Table S1) are highlighted by solid lines.

One bird tagged in FR left even earlier, on April 3rd, but made a long stop of 1.5 months at the Wadden Sea before resuming migration. Arrival at the breeding grounds occurred between the 26th of May and the 22nd of June, with the majority of birds arriving on the first half of June (Table 2). There were no major differences in the dates of the start and end of spring migration among birds tagged in the three wintering areas (Table 2). Dates of departure and arrival were positively correlated in spring (GLM, β = 0.711 ± 0.159, p < 0.001), meaning that earlier departing birds arrived at the breeding grounds earlier (Fig. 2, Supplementary Table S1). Departure date predicted migration speed (kms/day) in spring, with late plovers migrating faster that early ones (β = 3.652 ± 1.576, p < 0.05). This higher migration speed seems to result from a decrease in the total stopover duration rather than from a reduction in the number of stopovers (Table 2), although departure date did not predict total stopover duration nor the number of stopovers (Fig. 2, Supplementary Table S1).

**Figure 3:**
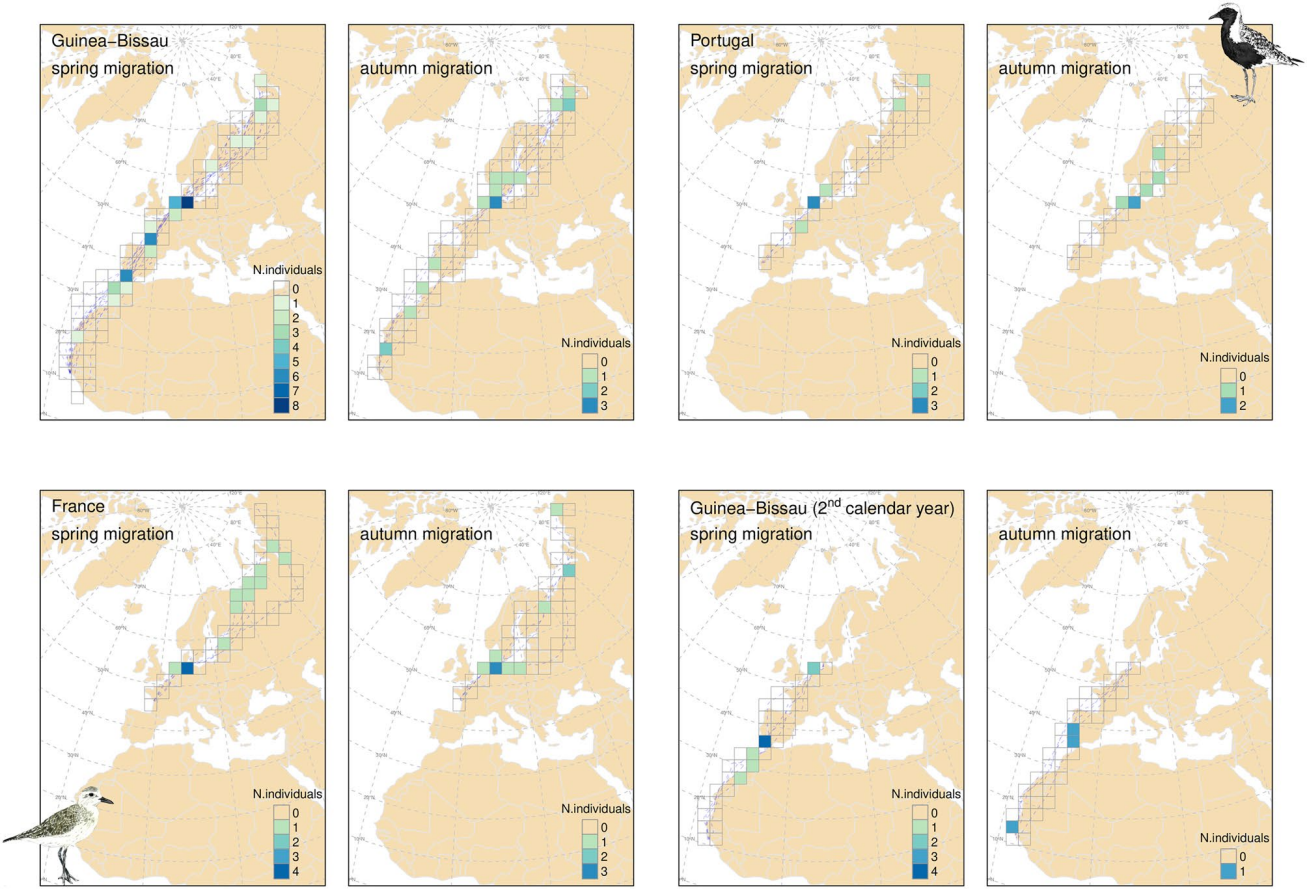
Relevance of stopover regions for migrating grey plovers during spring and autumn migration as determined by the number of individuals using each region. For this purpose, individuals tracked during two consecutive years were considered as two different individuals. The grid over the map is a regular square grid of 300 km. The basemap was obtained from R package rnaturalearth (version 0.1.0. https://CRAN.R-project.org/package=rnaturalearth), running in R software, which links to Natural Earth (https://www.naturalearthdata.com/) free vector maps.

Grey plovers departing from GB took a first long flight covering an average distance of 3498 ± 1125 km before stopping at least once along the African coast and/or in south Iberia. Next, they often stopped along the west coast of France (70% of the birds) before reaching the Wadden Sea (Fig. 3). All individuals used this area, stopping over either in The Netherlands, Germany or Denmark, and often making two or three consecutive stopovers (separated by more than 50km) within the region. Between the Wadden Sea and the breeding grounds, grey plovers did not show strong preferences for stopover sites (Fig. 3). Grey plovers wintering in PT and FR flew shorter first bouts, covering an average distance of 2488 ± 526 km and 1261 ± 236 km, respectively, and most individuals made a first stopover in the Wadden Sea (all birds stopped in this region). High proportions of birds from PT and FR were not found at any of the other stopover sites (Fig. 3). The average number of stopover sites used during spring migration was 4.7 for birds wintering in GB, while it was 2.7 for those wintering in PT and 3.8 for those wintering in FR. The duration of stopovers varied, with plovers wintering in GB, PT and FR stopping an average of 5.3, 7.4, and 6.8 days at each site, respectively (Table 2). Individuals often stopped for short periods, with one bird tagged in FR presenting up to 13 stationary periods (i.e., stops < 24h) in a single journey (Table 2). The average duration of spring migration was approximately one month and was similar among birds from different wintering sites, ranging from 18.8 to 62.2 days (Table 2). The mean distance travelled was 11,476 (± 431), 8094 (± 922) and 6411 (± 306) km for birds from GB, PT and FR, respectively. Migration speed was ca. 40% higher in birds from GB (355 ± 68 km/day) as compared to birds wintering in PT and FR (249 ± 27 and 242 ± 99 km/day, respectively; Table 2).

#### Autumn migration (adults)

The mean start of autumn migration was relatively synchronous for grey plovers wintering in Europe and Africa, although the departures from breeding areas spanned between 10 July and 23 August, probably reflecting differences in individual breeding success. In contrast, the arrival at the wintering grounds was highly variable (ranging from 16 August to 20 November), both among and within birds wintering in the three study areas (Table 2). Plovers heading towards more southern wintering areas arrived later, with birds reaching GB on average 1.5 to 2 months later than birds travelling to PT and FR (Table 2). During autumn migration we found no significant effects of departure date on the arrival date, migration speed, and the number and total duration of stopovers (Fig. 2, Supplementary Table S1). Average migration duration was two-fold longer for birds wintering in GB (83.1 ± 29.0 days) compared to PT (34.4 days) and FR (31.9 ± 7.4 days), and also more than twice as long as spring migration for the former group of birds (Table 2). In contrast to spring migration, autumn migration speed of plovers tagged in GB (142 ± 59 km/day) was ca. 40–50% slower than that recorded for PT and FR birds (200 and 213 ± 36 km/day, respectively; Table 2).

The average number of stopovers during autumn migration was similar to that used during spring and among sites (4.8 ± 1.3, 3.5 ± 0.7, 3.3 ± 1.5 for GB, PT and FR, respectively), ranging between two and six (Table 2). As in spring, all tracked individuals stopped at the Wadden Sea (mostly the German and Dutch regions). Before reaching this region, grey plovers used several stopover sites, notably the Khaypudyrskaya Bay (Pechora Sea) in Russia (approximately 60°E), and the Baltic Sea region (Fig. 3). Plovers heading to PT and FR did not stop (except one bird from PT that stopped once) between the Wadden Sea and their wintering areas, while plovers travelling to GB used a few stopover sites in Iberia or West Africa (Fig. 1). Two of the four birds wintering in GB for which we have complete autumn migration tracks, stopped at the Tagus estuary, in PT, and at the Diawling National Park, in Mauritania. Stopover duration was slightly longer during autumn than in spring, especially for birds travelling to GB (Table 2).

#### Second calendar year birds (non-breeders)

Second calendar year plovers travelled ca. 7000 km to and from the Wadden Sea, where they spent the summer. Departure date from and to the wintering grounds mostly overlapped with that of breeding birds, although on average the start of autumn migration was later in non-breeders (Table 2). Non-breeders used an average of 2.3 (± 0.5) stopover sites during spring migration, but this number was notably lower for autumn migration (0.7 ± 1.2), and two of the four tracked birds made a non-stop flight from the Wadden Sea to GB. As recorded for breeders during spring migration, 2nd calendar year plovers stopped at least once along the coast of Morocco, making further stops in Iberia and/or France. Mean stopover duration was similar for spring and autumn migrations (Table 2). Spring migration of 2nd calendar year birds lasted between 20 and 27 days, while autumn migration varied between less than 3.5 days and 21 days (Table 2).

#### Stopover sites

Tracked grey plovers used a large number of different stopover sites along their migratory routes. In total, we identified 66 sites which were used on average 2.1 (± 3.5) times (by the same individual or different individuals regardless of their breeding status and migration season). This corresponds to an average use of each site of 10% (± 20%), being the use of each site estimated as the proportion of bird trips that included that site as a stopover in relation to the total number of bird trips that crossed the same site (with the bird stopping or not; see Supplementary Table S2). Almost 70% of all stopover sites were used only once. Indeed, the rate of (new) stopover detection increased approximately linearly with the increasing number of tagged plovers, and the accumulation curves for new sites used by birds wintering both in GB and Europe (PT and FR) are almost linear (see Supplementary Fig. S2). This suggests that more new sites would have been detected, had we tracked more birds.

Overall, considering grey plovers from the different wintering grounds and regardless of their breeding status and migratory season, five stopovers were used in more than 20% of the recorded trips: three in the Wadden Sea (two in Germany and one in The Netherlands, n total = 31 trips), the Sidi Moussa-Walidia wetland complex in Morocco (n total = 26 trips) and the Khaypudyrskaya Bay (Pechora Sea) in Russia (n total = 34 trips; Supplementary Table S2). At a regional level, and apart from the Wadden Sea, the northwest of Morocco, south Iberia, western France, the Baltic Sea, the White Sea and Pechora Sea were used by a high proportion of plovers either in spring or autumn (Figs. 1 and 3).

**Figure 4:**
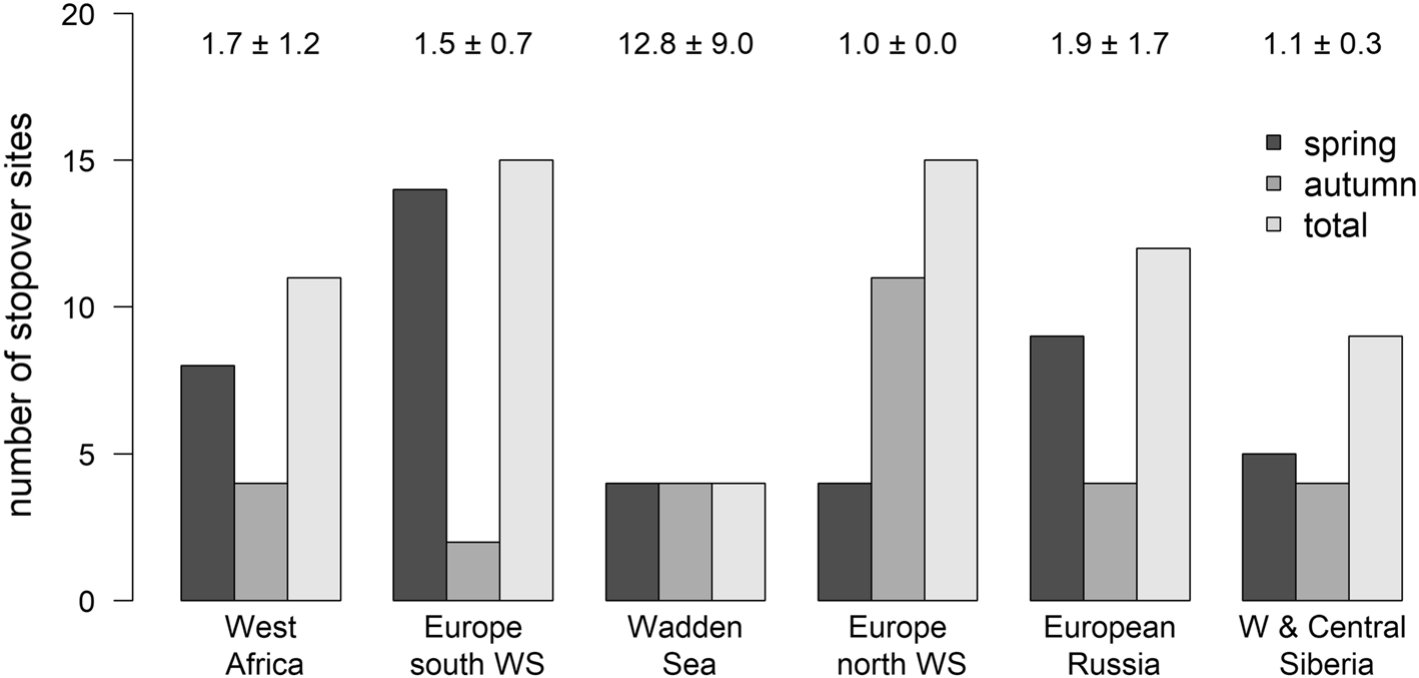
Number of stopover sites used by grey plovers during spring and autumn migration (and total) in six major geographic regions. The number over the bars represents the mean number of times each site was used by any bird in any migratory event.

The number of individuals stopping over (Fig. 3) and the number of stopover sites used (Fig. 4) varied considerably between spring and autumn migration in some geographic regions. The number of sites used in West Africa, Western Europe (i.e., Europe south of the Wadden Sea) and European Russia were two to six times higher in spring than in autumn (Fig. 4). Conversely, the number of sites used in North Europe (north of the Wadden Sea) during autumn migration was three times higher than in spring (Fig. 4).

**Figure 5:**
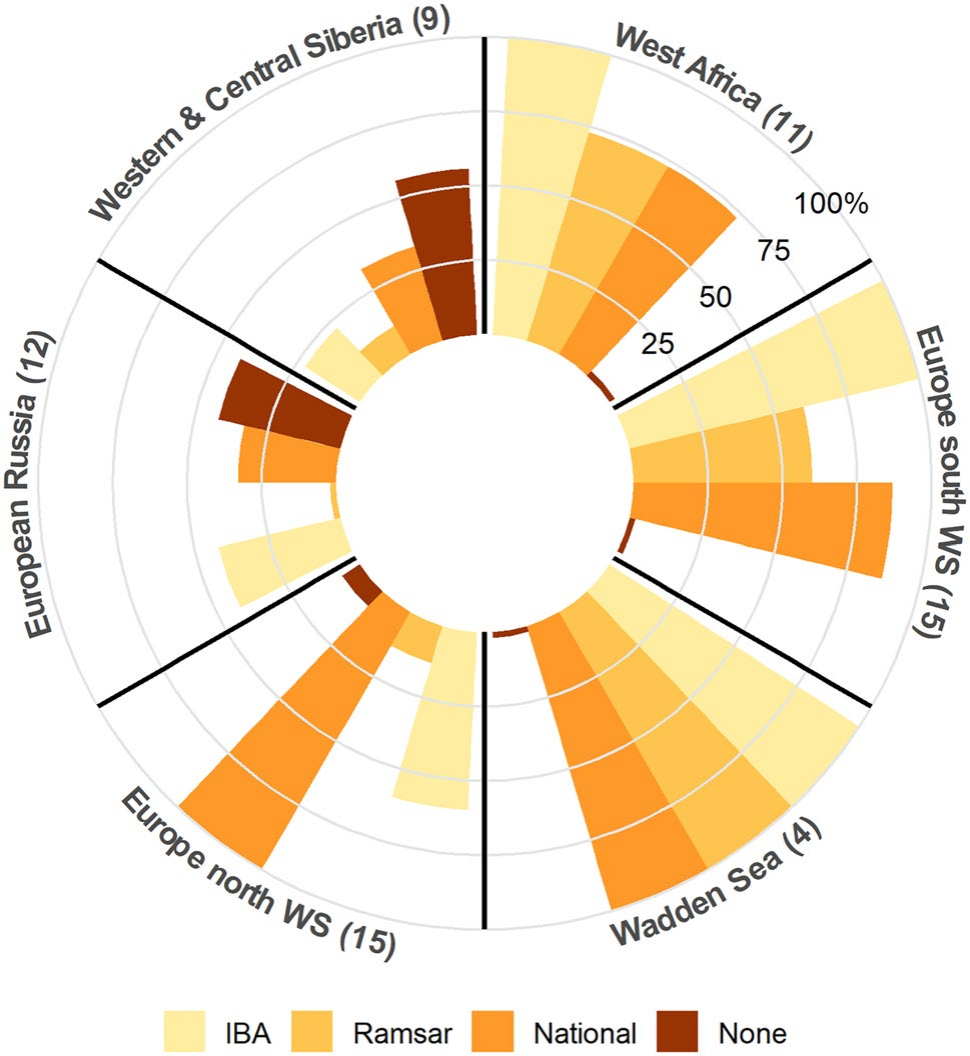
Protection status and classification as Important Bird and Biodiversity Area (IBA) of stopover sites used by grey plovers in the six large geographic regions encompassing their migratory routes. The total number of stopover sites identified in each region is presented in brackets.

**Figure 6:**
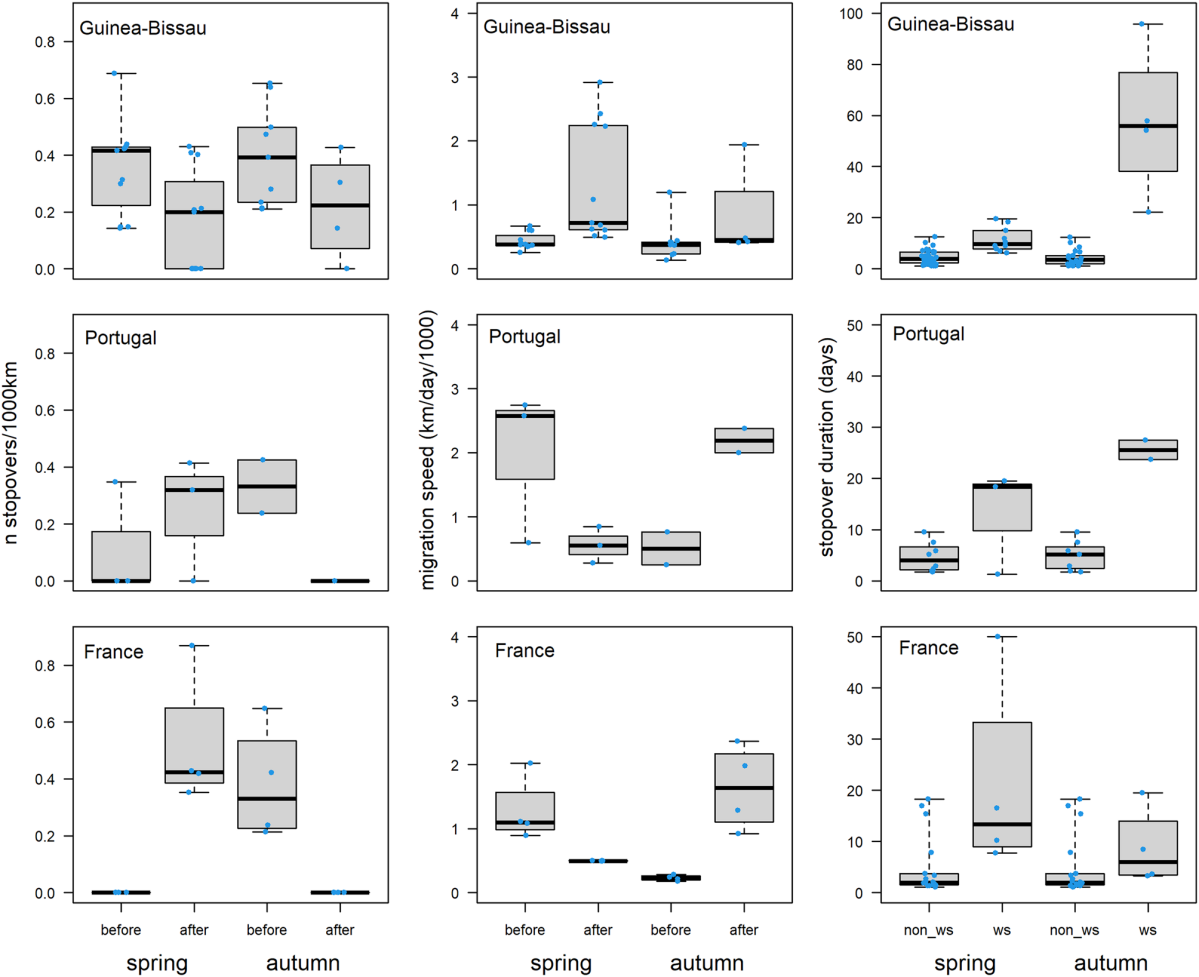
Number of stopovers (per 1000 km) and migration speed (km/day/1000) of grey plovers during spring and autumn migration before and after stopping at the Wadden Sea region, and duration (days) of stopover periods at the Wadden Sea (ws) and elsewhere (non_ws). Results are presented in separate panels for each wintering ground (Guinea-Bissau, Portugal and France). Different y-axis scales were used in the panel of stopover duration in Guinea-Bissau and those from Portugal and France.

The protection status and classification as Important Bird and Biodiversity Area (IBA) of stopover sites in six major geographic regions is presented in Fig. 6. Sites lacking any protection status or classification are virtually inexistent in most regions, except for the European Russia and Western and Central Siberia, where ca. 50% are not classified by either national or international authorities. In West Africa and Europe south of the Wadden Sea, all sites are classified IBA, over 60% are also designated as Ramsar sites, and more than 70% have a national protection status (either Natural Park, Natural Reserve or Special Protection Area; Fig. 5). In North Europe, all stopovers have a national protection status. In European Russia, the proportion of areas classified as IBA drops to 42%, and to 22% in Siberia (Fig. 5). From the 46 IBAs used by grey plovers, 19 have been attributed a threat score^39^, which for 75% of these sites is either “high” or “very high”.

#### Stopovers at the Wadden Sea

All tracked grey plovers used at least one stopover site within the Wadden Sea during both spring and autumn migration and birds stopped longer at the Wadden Sea than at any other site (Fig. 6). This difference in stopover duration was particularly noticeable during autumn for birds wintering in GB and PT, with mean stays at the Wadden Sea of 57.5 ± 30.1 days and 25.5 ± 2.7 days, respectively. Plovers wintering in FR spent less time at the Wadden Sea both in spring and autumn (21.1 ± 19.7 days and 8.7 ± 7.6 days, respectively. However, one individual departing early from FR stopped for 1.5 months at the Wadden Sea before resuming migration.

Grey plovers wintering in GB significantly increased migratory speed (km/day) after the stopover period at the Wadden Sea in both spring and autumn, which is also depicted by a lower number of stops from this point onwards (Fig. 6). In contrast, plovers from PT and FR always travelled fast between the Wadden Sea and their wintering grounds (Fig. 6), usually flying non-stop between these sites.

## Discussion

### Migration pattern and connectivity of the grey plover Eastern Atlantic population

Grey plovers wintering in GB, PT and FR bred mostly at Yamal Peninsula in Western Siberia, and to a lesser extent in European Russia, south of the Pechora Sea. One individual tagged in France bred further East, in Taimyr Peninsula, which has been previously recorded as a breeding ground of plovers wintering in Portugal and the Wadden Sea^25^. These results highlight a low migratory connectivity in the Eastern Atlantic population, with birds from the same breeding areas spreading to different regions during the non-breeding season. Yet, we have tracked a relatively small number of birds, and a larger sample would be ideal to quantify migratory connectivity strength as a metric^40^. Our results also show that grey plovers from Guinea-Bissau do not originate mostly from Central and possibly Eastern Siberia as it was previously suggested^24^. While our results do not support that Eastern Atlantic grey plovers exhibit a leap-frog migration pattern, as birds wintering at different latitudes strongly overlap at the European Russia and Western Siberia breeding areas, we believe that birds breeding in Central Siberia (mainly Taimyr) may consist of a considerably greater number of individuals from European wintering grounds. Indeed, none of the plovers tracked from Guinea-Bissau bred in such Eastern longitudes, but further tracking will be necessary to confirm these results.

### Migratory strategies: “skippers”, not “jumpers”

All tracked grey plovers followed a “skipping” migratory strategy, i.e., they flew mostly mid-distance bouts and used a network of stopover sites where they stopped usually for short periods^28^. The exception to this pattern is the long autumn fuelling period in the Wadden Sea of birds travelling to GB. These results contrast with the long-established idea that most Arctic breeding shorebirds that winter in intertidal habitats in West Africa (including the Red Knot, the Bar-tailed Godwit and the Grey Plover, among others) are “jumpers”, completing their migratory journeys in only two long non-stop flights, with a unique lengthy stop at the Wadden Sea for refuelling^1,15,26,27,41–43^. It has been suggested that species such as red knots and bar-tailed godwits would only make extra “emergency” stops when individuals deplete their energy stores before reaching the Wadden Sea (e.g. due to unfavourable wind conditions during flight^41,44^. However, a recent study that tracked the migration of bar-tailed godwits from Mauritania and GB showed that, during spring, a considerable proportion of birds stopped in Spain, Portugal or France before heading and stopping over in the Wadden Sea^45^, and most performed additional stops afterwards, in the Baltic coast and particularly in the West-Siberian Plain^45^. After breeding, godwits also stopped at least once, north of the Yamal Peninsula, before staging at the Wadden Sea and, from there, flew non-stop to West Africa^45^. Likewise, preliminary results of Red Knot tracking also show that these birds stop more often than previously reported, both before and after the refuelling period at the Wadden Sea (Jan van Gils, pers. comm.). Baltic dunlins (*Calidris alpina*) wintering in Mauritania were also recently shown to follow a “skipping” strategy in spring, while in autumn half of the birds were “skippers” and the other half were “jumpers”, flying non-stop from the Wadden Sea to their West African winter quarters (^46^; see also^47^). Therefore, “skipping” strategies may be considerably more common than previously thought, especially during spring migration.

Grey plovers have been shown to perform non-stop flights of over 7000 kms in the East Asian-Australasian Flyway^48^ and, in this study, three birds flew non-stop between the Wadden Sea and GB in autumn. Such ability for long non-stop flights would thus theoretically allow grey plovers to complete migration in only two flights, with a single stop in the Wadden Sea. So, why are grey plovers “skippers” rather than “jumpers”? During spring, grey plovers travelling between GB and the Wadden Sea show relatively low travel speeds, stopping on average 2.4 times to cover approximately 7000 km. This suggests that grey plovers do not reach optimal pre-migratory fuelling in GB, being forced to stop and refuel several times *en route*. Alternatively, they may have enough (temporal) leeway, avoiding enduring the stress of a very long flight, perhaps under unfavourable winds. The first hypothesis is supported by the low prey density in GB^49^, which might limit fuelling rates prior to departure. Indeed, harvestable biomass for grey plovers in the Bijagós Archipelago (GB) was estimated to be seven times lower than in the Tagus estuary, in Iberia^49^. Poor pre-migratory fuelling conditions in wintering West African resulted in longer fattening periods, later departures and/or lower body mass at departure in red knots and sanderlings (*Calidris alba*^50,51^). Still, both species seem to be able to skip West Africa during spring migration, flying directly to Europe^26,51^. This contrasts with grey plovers (this study), dunlins^46^, ringed plovers (*Charadrius hiaticula*^52^) and common sandpipers (*Actitis hypoleucos*^53^) for which the coast of Morocco provides important stopover areas for a high proportion of birds. Further research is needed to determine whether these stops are necessary, due to birds depleting their fuel loads before reaching Europe, or are facultative, depending for example on changes in wind direction or intensity. The fact that most of these birds jump over West Africa during autumn migration when travelling from resource-richer European sites, strongly suggests that low food availability at wintering African sites might indeed shape spring migratory patterns.

Most grey plovers also use a “skipping” strategy between the Wadden Sea and their Arctic breeding grounds, both in spring and autumn. During spring migration, birds from GB increased travel speed and decreased the number of stopovers per km travelled after passing the Wadden Sea, while the opposite pattern was recorded for plovers wintering in PT and FR. Whereas the Wadden Sea represents a high-quality refuelling site^41,50^ which could provide the opportunity for a last non-stop flight towards the breeding grounds, further stops along this course may be important to infer *en route* environmental conditions and therefore adequately schedule the arrival at breeding areas (e.g.^54^). During autumn, plovers from the three study sites showed lower travel speed and higher number of stops before arriving the Wadden Sea. Fuelling conditions at Arctic coastal sites close to the breeding grounds may not be sufficient to ensure a direct fly to the Wadden Sea, but at this time birds lose their breeding time-constraints. As previously mentioned, the long stop at the Wadden Sea in autumn allowed fuelling trips between approximately 3000 and 7200 km, i.e., the necessary to fly non-stop to the wintering areas.

We identified a variable, but in some cases high (up to 13 events during a single migration), number of stationary events (stopovers of very short duration, i.e., 0–24 h) with both PinPoint GPS Argos and GPS-UHF transmitters. The number of stationary periods was particularly high for plovers tracked with GPS-UHF transmitters collecting fixes every hour (average 8.3 ± 3.7), thus allowing a more accurate quantification of these events. The ecological significance of these short stops is difficult to ascertain, as our sample size is small, but this observation clearly suggest that birds also require additional, small wetlands (or other adequate habitat) along the entire migratory range. Despite the current advances in tracking technology, most research on shorebird migration in the EAF was conducted using geolocators (e.g.^46,52,53^) or satellite devices (e.g.^25,45,55^) which often provide locations with large time intervals (geolocators provide two locations per day and satellite devices have large periods off in the duty cycle) preventing the identification of stopovers of short duration^56^. The future use of GPS transmitters, recording fixes at a much higher frequency, may therefore bring new and unexpected information to the study of shorebird migration.

### Relying on a network of stopovers sites

Grey plovers used a large number of stopover sites during migration, spanning the West African, European and Russian/Siberian coasts. We identified 66 different stopover sites, and approximately 70% of these were used only once, potentially indicating high habitat availability for grey plovers at the flyway scale. The use of numerous sites may reduce competition for food resources, which can be particularly relevant in regions with mostly small wetlands (such as the coasts of Morocco and southern Iberia), potentially with more limited food resources and lower carrying capacity. Grey plovers have been often described as worm specialists at European wintering grounds^1,57,58^, but they are indeed quite plastic, and may show generalist habits and opportunistic foraging^59^. Dietary plasticity can also explain the versatility in choosing stopover areas, particularly in small areas or areas with low food resources.

Most tracking studies conducted in the EAF provide limited information on the exact locations of stopovers as well as on their relative importance. It is therefore difficult to assess whether most of the areas identified in this study are important stopover sites for other species. It is reassuring that 70% of all sites used by grey plovers are classified as IBAs, and this percentage reaches 100% in both West Africa and in Europe south of the Wadden Sea. In addition, apart from the European Russia and Siberia, a high proportion of sites is also designated as Ramsar site or/and has a national protection status. This scenario contrasts with that of the East Asian–Australasian Flyway, where a recent tracking study on great knots (*Calidris tenuirostris*) revealed an unexpectedly high number (92) of stopover sites, 63% of which were previously unknown and were lacking any conservation designation^60^. Protected areas, even if relatively small and only used during short periods of the life cycle, have proved to be key in the conservation of migratory waterbirds^61^. However, the classification of sites by itself, without some sort of management, does not guarantee protection. Indeed, 75% of the IBAs used by plovers and granted with a threat score, have received “high” and “very high” threat scores^39^.

The use of a high number of different stopover sites by the Eastern Atlantic population of grey plovers may represent an advantage, providing alternatives if one or more sites become unsuitable. However, there is an obvious bottleneck within this network. The Wadden Sea region, encompassing areas in The Netherlands and Germany (and to a much lesser extent in Denmark), seems to be an obligatory stop for grey plovers, both in spring and autumn migrations. Regardless of their wintering origin, grey plovers spent more time in the Wadden Sea than at any other stopover site. In addition, the Wadden Sea is the primary over-summering destination of 2nd calendar year birds. The ongoing deterioration of habitat conditions in the Dutch Wadden Sea were shown to be responsible for major declines in red knots^10,11^, and the mechanical harvesting of lugworms^62,63^ is contributing for lower survival rates of bar-tailed godwits^15^. The effects of such changes on grey plovers are yet unknown, but potential impacts suffered in the Wadden Sea may affect the whole Eastern Atlantic population.

### Explaining contrasting trends of African and European wintering populations: cues from migratory strategies?

Our results point to a low migratory connectivity in the Grey Plover Eastern Atlantic population, with birds breeding in European Russia and Western Siberia spending the winter in both GB and Western Europe. Deterioration of conditions in the Arctic breeding areas are therefore unlikely to explain the contrasting trends recorded in African and European wintering populations. Likewise, migratory routes in Europe and Russia and spring arrival dates at the breeding grounds are similar among studied populations. Grey plovers used a high number of wetlands as stopover sites in Africa, Europe and Siberia, with most sites receiving a very low proportion of tracked birds. A site acting as a bottleneck is the Wadden Sea, but no differences were found in the use of this region by plovers from the three wintering sites. Still, habitat loss and deterioration of foraging conditions in the Wadden Sea^10,11,62,64^ may have more detrimental impacts in more constrained birds. During spring, grey plovers from GB, PT and FR stop for similar periods in the Wadden Sea (less than two weeks). Despite facing a comparable journey from here towards their breeding grounds, plovers from GB have already flown approximately 4300 to 5600 km more than birds from PT and FR, respectively, and may thus have higher energy demands. Previous studies on staging bar-tailed godwits in the Wadden Sea have shown that Afro-Siberian and European populations have similar intake rates, but Afro-Siberian birds achieve higher fuel loads by foraging for longer periods per day^41^. Spending more time foraging comes at the expense of other maintenance activities such as resting and vigilance, with potential impacts on fitness and/or survival^65,66^. In response to the advancing springs in Siberia, Afro-Siberian bar-tailed godwits have shortened their refuelling period in the Wadden Sea, which reduced adult survival, especially in years of low food availability^15^. Although no data is available for grey plovers, as an Arctic breeding species that also feeds on worms at temperate latitudes^1,57,58^ they may face similar constrains. Plovers wintering in GB, with likely higher energy demands, may be more severely affected than their European counterparts.

Compared to individuals wintering in Europe, grey plovers from GB rely on stopover sites along the coasts of Morocco, Iberia and France, particularly during spring migration. Despite the diversity of sites used, the number of coastal wetlands in West Africa is limited, and most of the sites used in Morocco are seriously threatened by human activities, including the exploitation of water and drainage schemes for agricultural projects, pollution from pesticide and fertilizer run-off from agriculture, illegal hunting, and tourist development^39^. Yet, trends of wintering shorebirds in Morocco have changed little during the last decades^6^, providing little support for a strong deterioration in conditions for migrating grey plovers. While longer migrations usually entail greater risks, especially for birds crossing areas with fewer stopover opportunities^67^, potential mortality of tracked grey plovers (if using loss of transmitter signal as a proxy) was not associated to this geographic area.

## Conclusions

This study represents the first attempt to use tracking data of shorebirds from different wintering areas across the EAF to link migratory strategies with the contrasting population trends recorded in Europe and Africa. Although our study raises some hypotheses on the potential impact of harsher migratory conditions of grey plovers wintering in GB, we still lack evidence to establish a causal link between migratory strategies and population trends. Our results also show that grey plover populations wintering in Europe and West Africa are strongly mixed at breeding areas, thus hardly supporting any hypothesis relating divergent trends with the use of particular breeding grounds. Given that conditions in GB have not significantly changed during the last decades, another potential explanation may be a distributional shift in response to a warming winter climate, especially in Northwest Europe, which would also enable a shorter travelling distance to the breeding grounds^6^.

### Supplementary Information

Supplementary Information.

## Data Availability

The datasets generated during and/or analysed during the current study are available from the corresponding author on reasonable request. More information on the datasets can be found in Movebank: LIMITRACK [ID PROG 366]: https://www.movebank.org/cms/webapp?gwt_fragment=page=studies,path=study325569416. Migration of Grey Plover (*Pluvialis squatarola*): https://www.movebank.org/cms/webapp?gwt_fragment=page=studies,path=study1019418493.
