# Supplementary material for: Low migratory connectivity and similar migratory strategies in a shorebird with contrasting wintering population trends in Europe and West Africa
Source: Sci Rep. 2024 Feb 28;14:4884. doi: 10.1038/s41598-024-55501-y (PMC10901768; doi:10.1038/s41598-024-55501-y)

**SUPPLEMENTARY MATERIAL**

**Low migratory connectivity and similar migratory strategies in a shorebird with contrasting wintering population trends in Europe and West Africa**

Teresa Catry, Edna Correia, Jorge S. Gutiérrez, Pierrick Bocher, Frédéric Robin, Pierre Rousseau, José P. Granadeiro

^1^Centro de Estudos do Ambiente e do Mar (CESAM), Departamento de Biologia Animal, Faculdade de Ciências da Universidade de Lisboa, 1749-016, Lisboa, Portugal

^2^Departamento de Anatomía, Biología Celular y Zoología, Facultad de Ciencias, Universidad de Extremadura, Badajoz, Spain

^3^Ecología en el Antropoceno, Unidad asociada CSIC-UEX, Universidad de Extremadura, Badajoz, Spain.

^4^Laboratory Littoral Environnement et Sociétés UMR LIENSs 7266 CNRS-La Rochelle University, La Rochelle, France

^5^Ligue pour la Protection des Oiseaux (LPO), Rochefort, France

^6^National Nature Reserve of Möeze-Oléron, Ligue pour la Protection des Oiseaux (LPO), Saint-Froult, France

* Corresponding author: [teresa.catry@gmail.com](mailto:teresa.catry@gmail.com)

**Supplementary Table S1.** Generalized linear models assessing the effect of migration departure date and wintering origin of grey plovers on arrival date, migration speed (kms/day), number of stopovers and total duration spend on stopover. Model selection was performed by starting with the full model and comparing increasingly simple nested models, successfully removing non-significant predictors and interactions, with likelihood ratio tests (F-tests). The table presents the full model and the selected final model with the respective AIC values and the coefficients (estimate) and associated standard errors (SE) for the final model. t-tests and corresponding p-values are presented to show the significance of each term in the final model.

| Season |  | AIC | Estimate | SE | t-value | p-value |
| --- | --- | --- | --- | --- | --- | --- |
| Spring | Full model:  Arrival date ~ Departure date*Wintering origin | 103.27 |  |  |  |  |
|  | Final model:  Arrival date ~ Departure date+Wintering origin | 102.19 |  |  |  |  |
|  | Intercept |  | 71.118 | 20.478 | 3.473 | 0.0041 |
|  | Departure date |  | 0.711 | 0.159 | 4.466 | 0.0006 |
|  | Wintering origin (GB=0, Europe=1) |  | -7.500 | 2.708 | -2.769 | 0.0159 |
|  |  |  |  |  |  |  |
| Autumn | Full model:  Arrival date ~ Departure date*Wintering origin | 93.92 |  |  |  |  |
|  | Final model:  Arrival date ~ Departure date+Wintering origin | 92.12 |  |  |  |  |
|  | Intercept |  | 26.158 | 129.61 | 0.202 | 0.8458 |
|  | Departure date |  | 1.265 | 0.602 | 2.101 | 0.0737 |
|  | Wintering origin (GB=0, Europe=1) |  | -48.061 | 12.649 | -3.800 | 0.0067 |
|  |  |  |  |  |  |  |
| Spring | Full model:  Migration speed ~ Departure date*Wintering origin | 177.46 |  |  |  |  |
|  | Final model:  Migration speed ~ Departure date+Wintering origin | 175.57 |  |  |  |  |
|  | Intercept |  | -117.99 | 202.85 | -0.582 | 0.5707 |
|  | Departure date |  | 3.652 | 1.576 | 2.317 | 0.0375 |
|  | Wintering origin (GB=0, Europe=1) |  | -75.524 | 26.828 | -2.815 | 0.0146 |
|  |  |  |  |  |  |  |
| Autumn | Full model:  Migration speed ~ Departure date*Wintering origin | 100.94 |  |  |  |  |
|  | Final model:  Migration speed ~ Wintering origin | 98.08 |  |  |  |  |
|  | Intercept |  | 147.69 | 22.85 | 6.463 | 0.0003 |
|  | Wintering origin (GB=0, Europe=1) |  | 63.08 | 30.66 | 2.058 | 0.0786 |
|  |  |  |  |  |  |  |
| Spring | Full model:  Number stopovers ~ Departure date*Wintering origin | 68.72 |  |  |  |  |
|  | Final model:  Number stopovers ~ Wintering origin | 64.93 |  |  |  |  |
|  | Intercept |  | 1.548 | 0.146 | 10.610 | 2e-16 |
|  | Wintering origin (GB=0, Europe=1) |  | -0.358 | 0.255 | -1.407 | 0.159 |
|  |  |  |  |  |  |  |
| Autumn | Full model:  Number stopovers ~ Departure date*Wintering origin | 42.75 |  |  |  |  |
|  | Final model:  Number stopovers ~ 1 (null model) | 38.38 |  |  |  |  |
|  | Intercept |  | 1.361 | 0.160 | 8.499 | 2e-16 |
|  |  |  |  |  |  |  |
| Spring | Full model:  Total stopover duration ~ Departure date*Wintering origin | 99.47 |  |  |  |  |
|  | Final model:  Total stopover duration ~ Departure date+Wintering origin | 98.58 |  |  |  |  |
|  | Intercept |  | 62.991 | 18.293 | 3.444 | 0.0044 |
|  | Departure date |  | -0.287 | 0.142 | -2.020 | 0.0645 |
|  | Wintering origin (GB=0, Europe=1) |  | -5.842 | 2.419 | -2.415 | 0.0312 |
|  |  |  |  |  |  |  |
| Autumn | Full model:  Total stopover duration ~ Departure date*Wintering origin | 86.87 |  |  |  |  |
|  | Final model:  Total stopover duration ~ Wintering origin | 83.03 |  |  |  |  |
|  | Intercept |  | 76.300 | 9.905 | 7.703 | 0.0001 |
|  | Wintering origin (GB=0, Europe=1) |  | -48.500 | 13.289 | -3.650 | 0.0082 |

**Supplementary Table S2.** List of stopover sites used by grey plovers during spring and autumn migration. The number of trips refer to all the trips that passed on each site and could thus be translated into a potential stop. The proportion of use is the ratio between the number of stops and the number of trips. The protection/calssification status referred as “National” includes classifications as Special Protection Area (SPA, Birds Directive), National Park and Natural Reserve.

| Region | Site | Migration season | Number of stops | Number of trips | Prop. of use | Stopover duration (days) | Protection/  Classification Status |
| --- | --- | --- | --- | --- | --- | --- | --- |
| West Africa | Delta du Saloum National Park, Senegal | autumn | 1 | 8 | 0.13 | 5.6 | IBA,  Ramsar, National |
|  | Diawling National Park, Mauritania | autumn | 2 | 9 | 0.22 | 7.3 ± 1.8 | IBA,  Ramsar, National |
|  | Banc d'Arguin National Park, Mauritania | spring | 1 | 17 | 0.06 | 1.3 | IBA,  Ramsar, National |
|  | Canary current shelf, Western Sahara | spring | 1 | 17 | 0.06 | 10.7 | IBA |
|  | Khenifiss National Park, Morocco | autumn | 1 | 9 | 0.11 | 2.1 | IBA,  Ramsar, National |
|  | Souss-Massa and Aglou National Park, Morocco | spring | 2 | 17 | 0.12 | 7.8 ± 1.3 | IBA,  Ramsar, National |
|  | Tamri and Imsouane, Morocco | spring | 1 | 17 | 0.06 | 10.6 | IBA |
|  | Sidi Moussa-Oualidia, Morocco | spring | 4 | 17 | 0.24 | 12.6 ± 2.5 | IBA,  Ramsar |
|  |  | autumn | 1 | 9 | 0.11 | 4.4 |  |
|  | Merja Zerga, Morocco | spring | 1 | 17 | 0.06 | 6.8 | IBA,  Ramsar, National |
|  | Marais du Bas Loukous/Marais Larache, Moroccco | spring | 2 | 17 | 0.12 | 8.8 ± 7.3 | IBA, National |
|  | Oued Tahadart, Morocco | spring | 2 | 17 | 0.12 | 7.0 ± 4.2 | IBA,  Ramsar, National |
| Europe south of the Wadden Sea | Cadiz Bay, Spain | spring | 1 | 17 | 0.06 | 7.1 | IBA,  Ramsar, National |
|  | Doñana National Park, Spain | spring | 1 | 17 | 0.06 | 2.3 | IBA,  Ramsar, National |
|  | Marismas del Odiel, Spain | spring | 1 | 17 | 0.06 | 5.2 | IBA,  Ramsar, National |
|  | Isla Cristina and Ayamonte marshes and Prado lagoon, Spain | autumn | 1 | 9 | 0.11 | 11.9 | IBA,  National |
|  | Castro Marim and Vila Real de Santo António Natural Reserve, Portugal | spring | 1 | 9 | 0.11 | 3.2 | IBA,  Ramsar, National |
|  | Ria Formosa Natural Park, Portugal | spring | 2 | 17 | 0.12 | 5.6 ± 2.8 | IBA,  Ramsar, National |
|  | Tagus Estuary Natural Reserve, Portugal | Spring | 1 | 17 | 0.06 | NA | IBA,  Ramsar, National |
|  |  | autumn | 2 | 9 | 0.22 | 9.5 ± 4.1 |  |
|  | Arcachon Bay, France | spring | 2 | 17 | 0.12 | 4.4 ± 0.7 | IBA,  Ramsar, National |
|  | La Gironde Estuary, France | spring | 2 | 17 | 0.12 | 3.4 | IBA,  National |
|  | Aiguillon Bay Nature Reserve, France | spring | 3 | 17 | 0.18 | 5.0 ± 1.0 | IBA,  National |
|  | Moêze-Oléron Nature Reserve, France | spring | 1 | 17 | 0.06 | 2.8 | IBA,  National |
|  | Rade de Brest: Baie de Daoulas et Anse du Poulmic, France | spring | 1 | 16 | 0.06 | 2.4 | IBA,  National |
|  | Orne estuary, France | spring | 1 | 16 | 0.06 | 2.4 | IBA,  National |
|  | Westerschelde and Saeftinghe, The Netherlands | spring | 1 | 16 | 0.06 | 4.2 | IBA,  Ramsar, National |
|  | Delta Coast/Voordelta, The Netherlands | spring | 1 | 16 | 0.06 | 1.7 | IBA,  Ramsar, National |
| Wadden  Sea | Lower Saxony Wadden Sea National Park, Germany | spring | 9 | 16 | 0.56 | 5.4 ± 6.4 | IBA,  Ramsar, National |
|  |  | autumn | 3 | 15 | 0.20 | 1.6 |  |
|  | Wadden Sea, The Netherlands | spring | 10 | 16 | 0.63 | 13.7 ± 16.4 | IBA, Ramsar, National |
|  |  | autumn | 3 | 15 | 0.20 | 21.8 ± 3.3 |  |
|  | Schleswig-Holstein Wadden Sea National Park, Germany | spring | 13 | 16 | 0.81 | 9.2 ± 4.5 | IBA, Ramsar, National |
|  |  | autumn | 11 | 15 | 0.73 | 31.0 ± 30.3 |  |
|  | Vadehavet, Wadden Sea, Denmark | spring | 1 | 16 | 0.06 | 8.7 | IBA, Ramsar, National |
|  |  | autumn | 1 | 15 | 0.07 | 2.2 |  |
| Europe north of the Wadden Sea | Hardangervidda, Norway | autumn | 1 | 15 | 0.07 | 5.1 | IBA, National |
|  | Roskilde Fjord, Denmark | autumn | 1 | 15 | 0.07 | 1.5 | National |
|  | Horsens Fjord, Svanegrunden and Endelave, Denmark | spring | 1 | 19 | 0.05 | 5.8 | IBA, Ramsar, National |
|  | Pärnu bay, Estonia | spring | 1 | 19 | 0.05 | 1.1 | IBA, National |
|  | Signiskar - Market, Finland | spring | 1 | 19 | 0.05 | 1.8 | National |
|  | Boxo, Finland | autumn | 1 | 15 | 0.07 | 5.2 | National |
|  | Saaristomeren kansallispuisto/Archipelago National Park, Finland | spring | 1 | 19 | 0.05 | 1.3 | National |
|  | Northwest Stockholm (Gräddö) | autumn | 1 | 15 | 0.07 | 4.2 | None |
|  | Bay of Brå – Hävringe Island, Sweden | autumn | 1 | 15 | 0.07 | 6.5 | IBA, National |
|  | Eastern Gotland, Sweden | autumn | 1 | 15 | 0.07 | 1.9 | IBA, National |
|  | Holmöarna Archipelago, Sweden | autumn | 1 | 15 | 0.07 | NA | IBA, National |
|  | Oland, Sweden | autumn | 1 | 15 | 0.07 | 5.1 | National |
|  | Kalmar – Pataholm, Sweden | autumn | 1 | 15 | 0.07 | 3.6 | IBA |
|  | Vistula river mouth, Poland | autumn | 1 | 15 | 0.07 | 3.7 | IBA, National |
|  | Słowiński National Park Poland | autumn | 1 | 15 | 0.07 | 3.4 | IBA, Ramsar, National |
| European Russia | Terski coast, Russia | spring | 1 | 19 | 0.05 | 2.2 | IBA |
|  | Onega Bay of the White Sea, Russia | spring | 2 | 19 | 0.11 | 3.0 ± 0.4 | IBA |
|  | Onezhskoe Pomor'e, Russia | autumn | 2 | 15 | 0.13 | 12.5 ± 8.2 | National |
|  | White Sea mouth, Russia | spring | 1 | 19 | 0.05 | 1.4 | None |
|  | South White Sea, Russia | spring | 1 | 19 | 0.05 | 1.1 | None |
|  | Kanin peninsula (watershed of Yazhma and Nyes' rivers), Russia | spring | 1 | 19 | 0.05 | 2.2 | IBA |
|  | Russki Zavorot Peninsula and eastern Malozemelskaya Tundra, Russia | autumn | 2 | 15 | 0.13 | 16.2 ± 1.2 | IBA, National |
|  | South Zavorot Peninsula, Russia | spring | 2 | 19 | 0.11 | 1.4 ± 0.3 | National |
|  | Cheshskaya Bay, Russia | spring | 2 | 19 | 0.11 | 2.9 ± 1.4 | None |
|  | Pechora tundra | spring | 1 | 19 | 0.05 | 2.9 | None |
|  | Khaypudyrskaya Bay, Russia | spring | 4 | 19 | 0.21 | 2.3 ± 0.5 | IBA, National |
|  |  | autumn | 3 | 15 | 0.20 | 10.0 ± 8.9 |  |
|  | Novaya Zemlya, Russia | autumn | 1 | 15 | 0.07 | 6.8 | None |
| Western and Central Siberia | Lower Yuribey, Yamal Peninsula, Russia | autumn | 1 | 15 | 0.07 | 9.4 | IBA, National |
|  | South Yamal Peninsula, Russia | spring | 1 | 19 | 0.05 | 1.3 | None |
|  | Southeast Yamal Peninsula, Russia | spring | 1 | 19 | 0.05 | 7.5 | None |
|  | Lower Ob', South Yamal Peninsula, Russia | spring | 1 | 19 | 0.05 | 5 | IBA, Ramsar |
|  | Central Yamal Peninsula, Russia | spring | 1 | 19 | 0.05 | 1.9 | None |
|  | Coastal central Yamal Peninsula, Russia | autumn | 2 | 15 | 0.13 | 2.7 ± 0.4 | National |
|  | Yamal' skij, North Yamal Peninsula, Russia | autumn | 1 | 15 | 0.07 | 8.4 | National |
|  | East Yamal Peninsula, Russia | spring | 1 | 1 | 1.00 | 1.3 | None |
|  | North Taimyr, Russia | autumn | 1 | 1 | 1.00 | 7.8 | None |

**Supplementary Figure S2.** Accumulated number of stopover sites recorded with increasing number of tracked grey plovers wintering in Guinea-Bissau and Europe (Portugal and France). The shaded regions represent ±2SD.


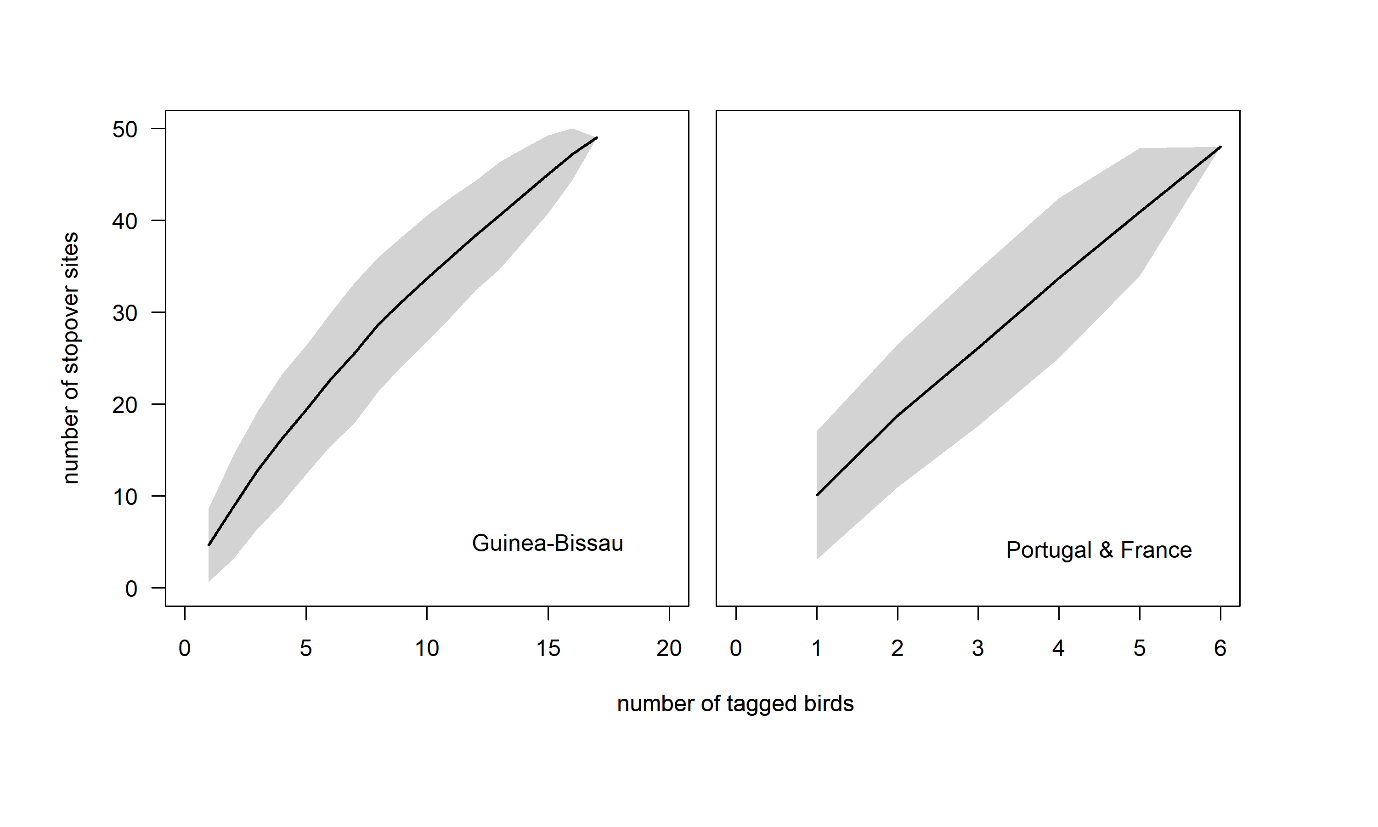

Supplement: Supplementary file 1 — Supplementary Information. [file 41598_2024_55501_MOESM1_ESM.docx]
